# Gemini: memory-efficient integration of hundreds of gene networks with high-order pooling

**DOI:** 10.1093/bioinformatics/btad247

**Published:** 2023-06-30

**Authors:** Addie Woicik, Mingxin Zhang, Hanwen Xu, Sara Mostafavi, Sheng Wang

**Affiliations:** Paul G. Allen School of Computer Science and Engineering, University of Washington, Seattle, WA 98195, United States; Paul G. Allen School of Computer Science and Engineering, University of Washington, Seattle, WA 98195, United States; Paul G. Allen School of Computer Science and Engineering, University of Washington, Seattle, WA 98195, United States; Paul G. Allen School of Computer Science and Engineering, University of Washington, Seattle, WA 98195, United States; Paul G. Allen School of Computer Science and Engineering, University of Washington, Seattle, WA 98195, United States

## Abstract

**Motivation:**

The exponential growth of genomic sequencing data has created ever-expanding repositories of gene networks. Unsupervised network integration methods are critical to learn informative representations for each gene, which are later used as features for downstream applications. However, these network integration methods must be scalable to account for the increasing number of networks and robust to an uneven distribution of network types within hundreds of gene networks.

**Results:**

To address these needs, we present Gemini, a novel network integration method that uses memory-efficient high-order pooling to represent and weight each network according to its uniqueness. Gemini then mitigates the uneven network distribution through mixing up existing networks to create many new networks. We find that Gemini leads to more than a 10% improvement in $F_{1}$ score, 15% improvement in micro-AUPRC, and 63% improvement in macro-AUPRC for human protein function prediction by integrating hundreds of networks from BioGRID, and that Gemini’s performance significantly improves when more networks are added to the input network collection, while Mashup and BIONIC embeddings’ performance deteriorates. Gemini thereby enables memory-efficient and informative network integration for large gene networks and can be used to massively integrate and analyze networks in other domains.

**Availability and implementation:**

Gemini can be accessed at: https://github.com/MinxZ/Gemini.

## 1 Introduction

Biological networks can provide insights into the underlying mechanisms of human diseases (Chen et al. 2009, 2014a,b, 2015; Li and Patra 2010; Lee et al. 2011; Smedley et al. 2014; Wang et al. 2014; Leiserson et al. 2015; Xuan et al. 2015; Liu et al. 2017), cell differentiation (Bruex et al. 2012; Wang et al. 2020), and protein essentiality (Zotenko et al. 2008). As high-throughput functional genomic screens have become more accessible, many large-scale biological networks have been produced, including protein–protein interaction networks (Uetz et al. 2000; Gavin et al. 2002; Ho et al. 2002; Bouwmeester et al. 2004), genetic interaction networks (Tong et al. 2004), metabolic networks (Lu et al. 2019; Lim et al. 2020), disease networks (Barabási et al. 2011; Žitnik and Zupan 2015), and patient similarity networks (Wang et al. 2014). The experimentally derived networks capture valuable information about the underlying biological system. However, each individual network is noisy and incomplete. Network integration methods seek to de-noise the underlying biological patterns and pool information from different types of biological networks, which can contain heterogeneous information about the underlying biology based on study conditions and experimental measurement techniques, and thereby improve understanding of gene biology (Mitra et al. 2013). Importantly, some kinds of networks may be less common than others, such as those derived from functional screens with more expensive equipment. These rare networks may still contain high-quality genetic patterns that are informative for many biological prediction tasks.

Gene networks, where each vertex represents a gene, are one common type of biological network. Such gene networks can be derived from many different experimental sources, including genetic interaction (Tong et al. 2004), co-expression (The GTEx Consortium 2015), physical interaction (Li et al. 2017), and co-localization (Qin et al. 2021), among many others. These different types of experimental data show different patterns, which can enhance our biological understanding of genes (Sharan et al. 2005; Zotenko et al. 2008). In addition, gene networks are often very large, containing several thousand genes each. In this work, we focus on unsupervised network integration methods, which can be broadly applicable for many biological tasks without the need for retraining and which learn gene–gene relationships directly from the interactome, thereby avoiding a bias toward known, labeled genetic interactions (Cusick et al. 2005). Although unsupervised network integration is an active and prolific area of research, including matrix decomposition approaches (Wang et al. 2014; Cho et al. 2015, 2016), deep-learning approaches (Gligorijevic et al. 2018; Zhang et al. 2018; Peng et al. 2021; Forster et al. 2022), kernel-based approaches (Lanckriet et al. 2004; Tsuda and Noble 2004), and regression-based approaches (Mostafavi et al. 2008; Mostafavi and Morris 2010), many existing network integration methods cannot scale to hundreds of genome-scale gene networks. For example, the BioGRID (Oughtred et al. 2021) human gene network collection contains 895 gene networks, and jointly storing all network diffusion states (Cao et al. 2014, 2013) would require 2.77 TB of memory. Network integration methods must therefore be very memory-efficient in order to be applied to these data. The scalability challenge, while already a significant constraint for large network collections, is also likely to worsen, as improved high-throughput screening technology enables more measurements for more genes under more conditions. It is therefore critical that gene network integration methods be scalable to increasing numbers of networks and of genes.

Moreover, existing state-of-the-art unsupervised network integration methods for massive biological datasets, such as Mashup (Cho et al., 2015, 2016; Wang et al. 2015), assume that important biological signal for a given gene is evenly distributed across networks in the dataset. Although this may hold for smaller, expertly curated network collections, this is unlikely to extend to hundreds of networks and is not the case in large interactome datasets that measure diverse genetic properties (Vidal et al. 2011). Intuitively, different types of networks, such as co-expression and co-localization (Soler-Oliva et al. 2017), can encode different features of a gene. Furthermore, measurements taken from different experimental conditions, such as diseased versus healthy or *in vitro* versus *in vivo*, can also contain complementary information about the underlying genetic system. In cases where a type of biological evidence or studied condition is more expensive to experimentally measure, this could cause a data collection to have fewer networks derived from those experiments. This in turn would diminish the integrated gene-level signal from these rare experiment sources.

**Figure 1.**
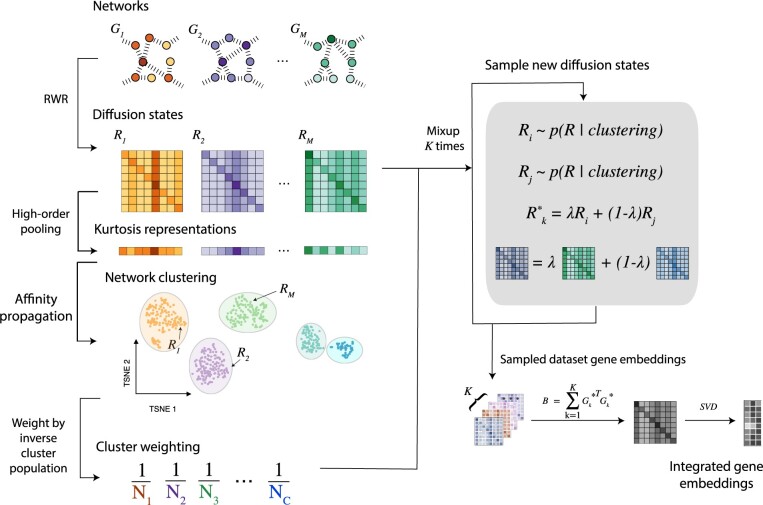
Overview of the Gemini algorithm. Given a set of input networks, Gemini uses random walk with restart to compute the diffusion states. Gemini then uses fourth-order kurtosis pooling of the diffusion state matrix as the feature vectors to cluster all networks. Gemini assigns each network a weight inversely proportional to its cluster size. It then randomly samples pairs of networks according to their weights. These pairs of diffusion state matrices are then mixed-up to create a new simulated network collection that more evenly covers the possible diffusion states. We finally aggregate the synthetic dataset and perform an efficient singular value decomposition to produce embeddings for all vertices in the network collection.

To address these limitations we present Gemini, an unsupervised network integration approach that has a memory complexity that is constant in the number of gene networks. Our key idea is to represent the diffusion state of each network as a vector using fourth-order kurtosis pooling. We then weight each network by an approximation of its uniqueness using this vector, which serves as a more memory-efficient surrogate for the full diffusion state representation. We then simulate many new networks by sampling and mixing-up original networks according to the uniqueness-based weights (Fig. 1). This simulation process enables users to either obtain more networks for a more robust integration or consider fewer representative networks for a shorter running time.

We evaluate Gemini on network-based protein function prediction, which has been extensively used to assess biological network integration methods (Lanckriet et al. 2004; Tsuda and Noble 2004; Sharan et al. 2005, 2007; Tsuda et al. 2005; Lee et al. 2006; Mostafavi et al. 2008; Voevodski et al. 2009; Mostafavi and Morris 2010; Cho et al. 2015, 2016; Wang et al. 2015; Peng et al. 2017, 2021; Gligorijevic et al. 2018; Singh et al. 2022). When integrating hundreds of human gene networks, we found that Gemini outperforms existing network integration methods by more than 10% in $F_{1}$ score, 15% in micro-AUPRC, and 63% in macro-AUPRC. Most importantly, we found that the quality of Gemini’s embeddings improved by 15% with respect to downstream micro-AUPRC when additional human networks were added to the dataset, while Mashup’s (Cho et al. 2016) performance degraded by 4%. Gemini can therefore balance the many different sources of biological information in the final embeddings while maintaining computational scalability, and can be broadly applied to networks both within and outside of biology for efficient integrative analysis.

## 2 Materials and methods

### 2.1 Problem definition

We define our input dataset of *M* networks as $G^{\left(M\right)}=\{G_{1},G_{2},\ldots ,G_{M}\}$, where all $G_{i}$ have the same *n* vertices and can be represented by their adjacency matrix $A_{i}$. Each network $G_{i}$ may or may not be weighted or directed. Gemini then outputs an embedding for each vertex, $Z\in R^{n\times d}$, where *d* is a user-defined embedding dimension, and the embedding $\vec{z_{j}}$ can be used as the feature vector for vertex *j* in downstream tasks.

### 2.2 Review of Mashup

Gemini’s skeleton is based on the memory-efficient version of Mashup (Cho et al. 2016), the state-of-the-art network integration approach with memory complexity that is constant in the number of networks and quadratic in the number of nodes, which is critical when integrating hundreds of large networks. Fundamentally, Mashup contains three important steps:

1. Compute the diffusion state for each network using random walk with restart (RWR);
2. Integrate the diffusion state for each network;
3. Decompose the integrated states with singular value decomposition (SVD).

In the first step, the diffusion state is calculated from each network’s transition matrix $T_{i}$ using the RWR algorithm, iteratively computing the state for vertex *j* in network *i* as where α is a user-specified restart probability and $\vec{e_{j}}$ is a standard basis vector, and $\vec{r_{ij}}[k]$ is the probability assigned to vertex *k* given starting state *j* in network *i*. For notational simplicity, we denote the RWR diffusion matrix for all vertices in network *i* as $R_{i}\in R^{n\times n}$.


(1) $$\begin{array}{c} {\vec{r_{ij}}}^{\left(t+1\right)}=(1-\alpha )T_{i}{}^{⊤}{\vec{r_{ij}}}^{\left(t\right)}+\alpha \vec{e_{j}}\\ \vec{r_{ij}}={\vec{r_{ij}}}^{\left(\infty \right)}\in R^{n}, \end{array}$$

In the second step, Mashup integrates the *M* diffusion state matrices with concatenation, with $P=[R_{1},\ldots ,R_{M}]\in R^{Mn\times n}$. In the third and final step, Mashup computes the embedding $\vec{z_{i}}\in R^{d}$ for the *i*th vertex as where $\mathrm{SV}D_{\text{trunc}}$ indicates the truncated SVD, and $\sigma_{i}$ is the *i*th singular value and $v_{i}$ is the *i*th right singular vector of the concatenated log-transformed RWR matrices. To reduce memory usage, Mashup uses the identity that the eigenvectors of $A^{⊤}A$ are the same as the right singular vectors of A. Therefore, rather than the $O(Mn^{2})$ memory requirement for (2), we can instead compute which can be summed one network at a time, thereby only requiring $O(n^{2})$ memory. Finally, Mashup takes $\vec{z_{i}}={\left(\lambda_{i}\right)}^{1/4}\vec{\nu_{i}}$, where $\vec{\nu_{i}}$ is the *i*th eigenvector of Q and $\lambda_{i}$ is the corresponding eigenvalue, to efficiently compute the *i*th vertex embedding.


(2) $$\begin{array}{cc} U\Sigma V=\mathrm{SV}D_{\text{trunc}}([\text{log}(R_{1}),\ldots ,\;\text{log}(R_{M})]) & \vec{z_{i}}=\sqrt{\sigma_{i}}\vec{v_{i}}, \end{array}$$

(3) $$\begin{array}{c} Q={\left[\text{log}(R_{1}),\ldots ,\;\text{log}(R_{M})\right]}^{⊤}[\text{log}(R_{1}),\ldots ,\;\text{log}(R_{M})]\\ =\sum_{i=1}^{M}\;\text{log}\;{\left(R_{i}\right)}^{⊤}\;\text{log}(R_{i}), \end{array}$$

Although this enables fast and scalable integration of large networks, the concatenation of all diffusion state matrices in the second step introduces several important limitations. First, Mashup evenly weights information from all networks during the integration step. When applied to increasingly large number of networks without extensive pre-processing and curation, redundant networks may therefore dominate the downstream embeddings. Second, the networks contained in the input dataset are unlikely to cover the set of possible and reasonable inputs. Therefore, further pooling information from different networks could be useful for further improving downstream vertex embeddings. We aim to address these limitations by refining the second step with Gemini. Gemini maintains the same first step and third step as Mashup.

### 2.3 Efficient network similarity calculation

Rather than equally combining all networks in the final vertex embedding computation, Gemini weights networks according to their uniqueness, while maintaining efficient memory usage for the large network collection. Mean squared error (MSE) is a widely-used similarity measurement metric. It can therefore be used to quantify the similarity of input networks $G_{p}$ and $G_{q}$ based on their diffusion states as


(4) $$\delta_{\text{mse}}(R_{p},R_{q})=||R_{p}-R_{q}|{|}_{F}^{2}.$$

However, in real-world gene network collections there may be too many large networks to run this computation efficiently. Instead, we use RWR to construct a probability distribution centered around the starting vertex, and then quantify the dispersion to each vertex according to its kurtosis, the fourth standardized moment. Compared to low-order pooling (e.g. mean and variance), kurtosis can better capture the tailedness of the diffusion distribution, which can help quantify both global- and local centrality of each vertex in the network, thereby compactly representing its structure. We hypothesize that high-order pooling yields a good approximation for $\delta_{\text{mse}}(R_{p},R_{q})$ in our real-world datasets, and chose a fourth-order kurtosis poooling. Specifically, we approximate $\delta_{\text{mse}}(R_{p},R_{q})$ with for j∈{1,…,n}. Using the kurtosis pooling, we then compute network similarity with $\delta_{\text{kurt}}(\cdot )$ in an O(n) space, rather than an $O(n^{2})$ space, enabling more network representations to be loaded in memory simultaneously and thereby speeding computation.


(5) $$\begin{array}{cc} \delta_{\text{kurt}}(R_{p},R_{q})= & ||\mathrm{kurt}(R_{p})-\mathrm{kurt}(R_{q})|{|}_{2}^{2}, \end{array}$$

(6) $$\mathrm{kurt}(R_{i})[j]=\frac{\frac{1}{n}\sum_{k=1}^{n}(\vec{r_{ik}}[j]-(\frac{1}{n}\sum_{l=1}^{n}\vec{r_{il}}[j]){)}^{4}}{(\frac{1}{n}\sum_{k=1}^{n}(\vec{r_{ik}}[j]-(\frac{1}{n}\sum_{l=1}^{n}\vec{r_{il}}[j]){)}^{2}{)}^{2}},$$

### 2.4 Network weighting according to uniqueness

To identify groups of redundant networks, we then run the affinity propagation clustering algorithm (Frey and Dueck 2007) on the kurtosis representations. This does not require a prespecified number of clusters, but instead computes the number of clusters *C* based on the data. Therefore, if the dataset is already uniformly distributed (all networks are sufficiently dissimilar), they will not be clustered together; however, if the input dataset contains redundancies then similar networks, as defined by (5), will be more likely to cluster together. This step assigns each network to one of the *C* clusters computed by the algorithm as


(7) $$\vec{c}\in {\left\{1,\ldots ,C\right\}}^{M}=\mathrm{affn}\_\mathrm{prop}(\mathrm{kurt}(R_{1}),\ldots ,\mathrm{kurt}(R_{M})).$$

Finally, using $N_{c}$ to denote the number of input networks assigned to cluster *c*, for all networks i∈{1,…,M} we use propensity weighting to define the sampling probability distribution


(8) $$p(i|\vec{c}[i]=c)=\frac{1}{CN_{c}}.$$

### 2.5 Diffusion state sampling with mixup

The second limitation that we sought to address is that the input network collections may be unevenly distributed. To address this, Gemini assumes that the diffusion states jointly parameterize a latent understanding of gene similarity. Specifically, Gemini uses the sampling distribution in (8) as an inverse probability weighting (Rosenbaum 1987). This leads to instances from different clusters to be sampled equally frequently in expectation. In order to construct a new dataset that can be tuned to the user’s computational resources, we then construct new examples using mixup augmentation (Zhang et al. 2017), simulating for λ∈[0,1] sampled uniformly-at-random. We repeat this process *K* times, where *K* is a hyperparameter defined by the user based on their computational resources, to produce the simulated dataset ${\tilde{R}}^{\left(K\right)}=\{{\tilde{R}}_{1},\ldots ,{\tilde{R}}_{K}\}$, which can better represent possible diffusion state matrices for the input data than the original *M* networks. Although larger values of *K* improve the robustness of $\tilde{R}$, smaller values of *K* enable users with limited computational resources to efficiently integrate input networks with a reduced network collection size.


(9) $$\begin{array}{c} G_{i},G_{j}∼p(G|\vec{c})\\ \tilde{R}=\lambda R_{i}+(1-\lambda )R_{j} \end{array},$$

Using the simulated dataset ${\tilde{R}}^{\left(K\right)}$, we then repeat memory-efficient embedding step in Section 2.2 from Mashup (Cho et al. 2016) to efficiently compute the vertex representations $\vec{z_{j}}$ for each vertex *j*. We linearly normalize $\vec{z_{j}}[d]\in [0,1]$ with min-max normalization (Patro and Sahu 2015). As Gemini’s simulated, mixed-up network collection more evenly covers the types of evidence included in the input networks, the vertex embeddings also contain a more even sampling of evidence for all downstream tasks.

### 2.6 Application to protein function prediction

Biological network integration has been shown to work well in settings where guilt-by-association (Schwikowski et al. 2000) applies, meaning that connected nodes in the network demonstrate similar phenotypes of interest (Menche et al. 2015). One such setting is protein function prediction, which has been used as an important application to validate network integration approaches (Lanckriet et al. 2004; Tsuda and Noble 2004; Tsuda et al. 2005; Lee et al. 2006; Mostafavi et al. 2008; Voevodski et al. 2009; Mostafavi and Morris 2010; Cho et al. 2015, 2016; Wang et al. 2015; Peng et al. 2017, 2021; Gligorijevic et al. 2018). We therefore evaluate Gemini’s ability to efficiently generate informative embeddings from a heterogeneous set of input networks using a protein function prediction task, classifying each vertex in a network to a subset of *G* possible functions.

Given the vertex embeddings $\vec{z_{i}}$, we predict the protein’s function $y_{i}\in {\left[0,1\right]}^{G}$ as where σ(⋅) represents the sigmoid function. We learn $f_{\theta }(\cdot )$ on a subset of the *n* protein embeddings learned by the unsupervised network integration methods, and evaluate the embedding quality based on the remaining proteins.


(10) $${\vec{y}}_{i}=\sigma (f_{\theta }({\vec{z}}_{i}))\in R^{G},$$

## 3 Results

### 3.1 Dataset, comparison approaches, and experimental settings

We separately consider integration tasks for mouse, human, and yeast datasets to demonstrate Gemini’s applicability to different species with different dataset and network sizes. Additional network statistics are provided in the Supplementary Information.

### 3.2 BioGRID biological networks

We download the processed mouse, human, and yeast BioGRID (Oughtred et al. 2021) gene networks from Mostafavi et al. (Warde-Farley et al. 2010). Each organism collection contains more than 400 networks (Supplementary Table S1).

### 3.3 STRING biological networks

We also use gene networks from the STRING dataset (Szklarczyk et al. 2019), where edges indicate an association between two genes based on co-expression data, experimental data, or curated datasets. We use the pre-processed networks from Mashup (Cho et al. 2016), where network edges are weighted in [0, 1] according to the probability of edge presence. Each organism has 6 STRING networks (Supplementary Table S1). Although the STRING database only contains 6 networks for each species, they are curated; in comparison, GeneMANIA’s BioGRID dataset has many more networks for each species, but they are also more noisy.

### 3.4 GOA protein function annotations

We used the Gene Ontology Annotation (GOA) database (Camon et al. 2004; Huntley et al. 2014) for the downstream protein function prediction task. The GOA dataset comprises *G* = (16 626, 21 655, 8387) total protein function labels for mouse, human, and yeast, respectively. To further investigate results, we can stratify Gene Ontology (GO) terms according to the three GO sub-ontologies: biological process (BP), molecular function (MF), and cellular component (CC).

### 3.5 Comparison models

We compare to the Mashup network integration model (Cho et al. 2016), which can scale to many hundreds of input networks with reasonable computational power. Importantly, we use the memory-efficient version of Mashup that computes integrated gene embedding features with the eigendecomposition in Section 2.2, rather than the learnable objective that cannot scale to our datasets. We also compare to BIONIC (Forster et al. 2022), which uses a graph attention network to produce integrated gene embeddings, for yeast networks as well as human and mouse networks in the STRING collection. Given the large number of networks and size of each network (Supplementary Table S1), BIONIC is unable to scale to the mouse and human BioGRID datasets. Finally, we compare to gene embeddings produced from the network collection’s average adjacency matrix, $\bar{A}=\frac{1}{M}\sum_{i=1}^{M}A_{i}$. Specifically, we consider the principal component analysis (Jolliffe 1986) decomposition of $\bar{A}$, the truncated SVD of $\bar{A}$, and Mashup decomposition applied to $\bar{A}$.

We use α=0.5 for the restart probability in (1) and an embedding dimension d=400 for mouse and human, and d=200 for yeast. During Gemini’s clustering stage, we use Affinity Propagation (Frey and Dueck 2007) with a damping factor of 0.875, which we found to converge empirically. We train the BIONIC embeddings using the model defaults wherever possible with respect to GPU memory; for other methods, hyperparameters shared by multiple models use the same configurations to facilitate method comparison. Further discussion of method hyperparameters is provided in the Supplementary Information. For each species’ experiment, we use 5-fold cross-validation, where 80% of the data are seen during training of each fold. We divide that 80% into a further 80% training and 20% validation set to select the best number of training epochs, use a batch size of 64, and terminate training when the validation loss has not achieved a new global minimum for 50 epochs. We use the same protein function prediction model in (10) for all network integration models, where $f_{\theta }(\cdot )$ is implemented as a multilayer perceptron with two hidden layers, with 200- and 100-hidden units, respectively, rectified linear unit activation functions, and trained with cross entropy loss.

### 3.6 Evaluation metrics

We evaluate the models’ embedding quality in terms of their test performance on the downstream protein function prediction task. Since this is an imbalanced classification problem, we consider maximum $F_{1}$, which is the $F_{1}$ score with the optimal probability threshold for the model, and area under the precision-recall curve (AUPRC), with both macro-AUPRC and micro-AUPRC.

### 3.7 Gemini improves downstream protein function prediction on BioGRID

**Figure 2.**
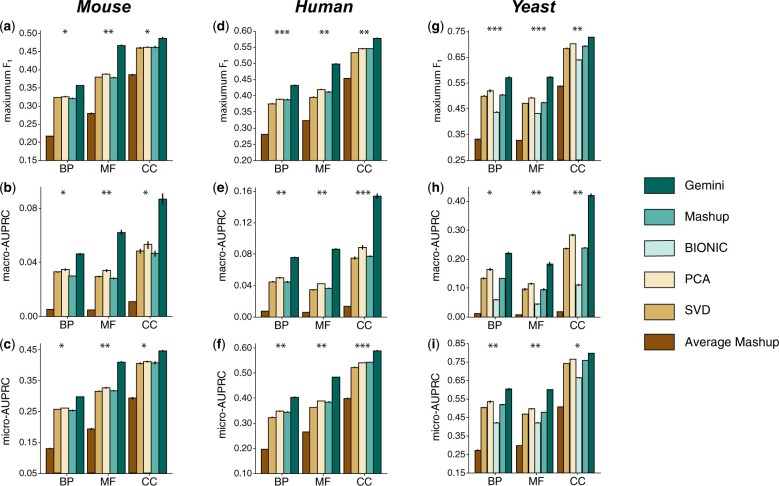
Performance of protein function prediction by integrating networks from BioGRID, stratified by GO sub-ontology. (a–i) Barplot of model performance for protein function prediction task, divided into the BP, MF, and CC sub-ontologies of GOA. Comparison of Mashup, BIONIC, Mashup on $\bar{A}$, principal component analysis on $\bar{A}$, SVD on $\bar{A}$, and Gemini on mouse (a–c), human (d–f), and yeast (g–i) species. We consider $F_{1}$ score (a, d, g), macro-AUPRC (b, e, h), and micro-AUPRC (c, f, i), where higher values indicate better performance and error bars indicate standard error over five test folds; one-sided paired *t*-test comparison with Mashup is also shown (*P-value < 5e−4, **P-value < 5e−5, and ***P-value < 5e−6).

We found that Gemini substantially outperformed the comparison approaches (Cho et al. 2015, 2016) on the BP, MF, and CC sub-categories of the GOA annotations for all three species (Fig. 2) by integrating hundreds of networks from BioGRID. Gemini has the largest improvement on human, where it has an average test $F_{1}$ score of 0.46, macro-AUPRC of 0.10, and micro-AUPRC of 0.45, compared to 0.42, 0.06, and 0.39 for the best-performing baseline, respectively. The mouse dataset also has a similar improvement, while the yeast improvement is significant but more modest. Since human and mouse networks are larger than yeast networks, this indicates the importance of weighting networks, especially in large network collections. Furthermore, we find that BIONIC and Mashup both struggle on this BioGRID network integration task; BIONIC’s Graph Convolutional Network framework may struggle with the relatively dense networks (Supplementary Table S1), while Mashup uses the network-specific weight in the SVD computation in (2) to model the more unique networks, rather than incorporating the information in the gene vertex embeddings. Gemini addresses these challenge by more evenly sampling different types and combinations of networks, thereby incorporating rare network information into the vertex embeddings as well as the network embeddings.

### 3.8 Gemini effectively integrates networks from BioGRID and STRING

We next evaluated Gemini’s performance on integrating all networks from BioGRID and STRING. We repeated the protein function classification task using the GOA database, comparing the quality of the embeddings learned from the STRING network collection, the BioGRID network collection, and the union of the STRING and BioGRID network collections. In such a setting, the union of the two network collections is a superset of the individual collection, and we therefore expect it to contain more information than either collection alone without removing any of the information. We would therefore hope that a network integration method would perform better on the combined network collections than either of the collections alone.

**Figure 3.**
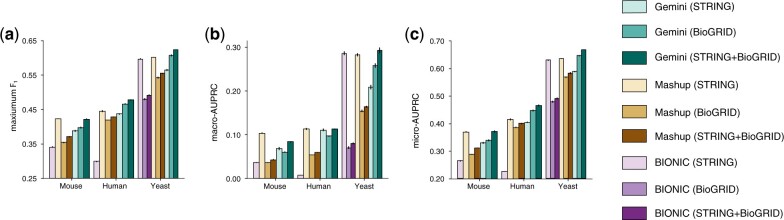
Comparison of network integration for BioGRID and STRING collections. (a–c) Barplots comparing Mashup, BIONIC, and Gemini network integration quality on the STRING, BioGRID, and union of STRING and BioGRID network collections, evaluated based on the average performance of the downstream protein function prediction task on GOA. BIONIC does not have BioGRID or STRING + BioGRID results for mouse or human due to GPU memory constraints. Results for mouse, human, and yeast experiments are shown. Test set performance is measured with the maximum $F_{1}$ score (a), macro-AUPRC (b), and micro-AUPRC (c). Higher values indicate better performance; errors bars indicate the standard error over five test folds.

We find that Gemini achieves peak performance on the combined dataset, indicating that it effectively makes use of all networks from two different sources (Fig. 3, Supplementary Fig. S2). In particular, Gemini has a maximum $F_{1}$ score of 0.62 on the combined yeast dataset, compared to 0.55 for Mashup. Furthermore, this improves compared to both Gemini’s $F_{1}$ score of 0.56 on the STRING and 0.61 on the BioGRID databases individually. As an important comparison point, we find that Mashup consistently performs best when embeddings are learned only from the STRING networks. We hypothesize that the curated STRING networks are all sufficiently diverse and high-quality that Gemini’s high-order pooling for network uniqueness and subsequent network-mixup are not helpful in this setting. However, Mashup’s downstream protein function annotation quality degrades when the BioGRID networks are also included. Specifically, while Gemini’s macro-AUPRC performance improves by more than 40% when yeast BioGRID networks augment the STRING input (one-sided *t*-test *P*-value < 5e−6 for improvement over STRING alone, *P*-value < 5e−3 for improvement over BioGRID alone), Mashup’s performance decreases by 71% (one-sided *t*-test *P*-value < 5e−9 compared to STRING alone). Furthermore, Gemini’s STRING + BioGRID integrated gene embeddings outperform Mashup’s STRING integrated embeddings, indicating the downstream benefit of additional, if noisy, networks. The prominent performance of Gemini when integrating hundreds of networks from many sources demonstrates Gemini’s wide applicability for integrating the accumulating and continually generated biological networks.

### 3.9 Kurtosis similarity reflects biological similarity

**Figure 4.**
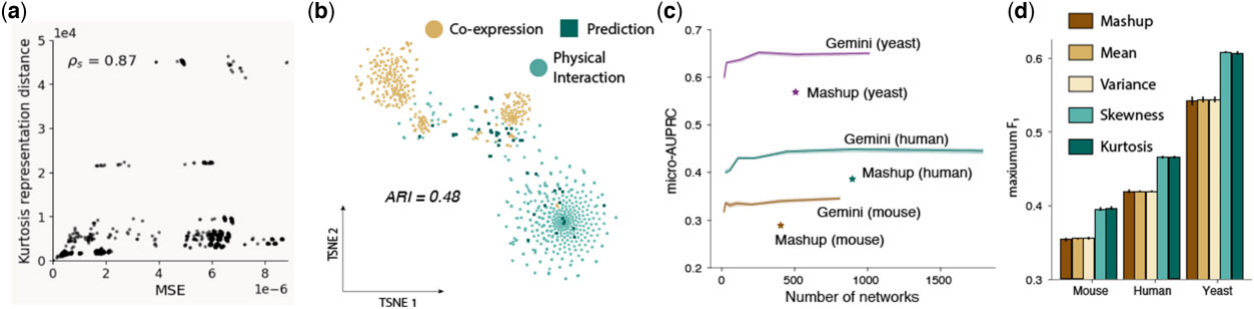
Gemini feature analysis on BioGRID networks. (a) Scatter plot of MSE- and kurtosis-based similarities for pairs of diffusion state matrices in the BioGRID yeast dataset, with a Spearman correlation of 0.87. (b) t-SNE embeddings of kurtosis vector representations for the BioGRID human dataset, colored by the type of biological evidence used to construct that network, with an ARI of 0.48. (c) The average macro-AUPRC (higher is better) with increasing numbers of mixed-up networks *K* for each species across 5 random seeds, compared to Mashup on the BioGRID dataset. (d) Barplot of downstream protein prediction $F_{1}$ score (higher is better) for mean-, variance-, skewness-, and kurtosis-based network similarity approximations, with Mashup included as a reference, on the BioGRID networks. Error bars indicate standard error over five test folds.

Finally, we sought to further understand the promising performance of Gemini by validating its hypotheses and network representations. We used $\delta_{\text{kurt}}$ in (6) to approximate the slower-to-compute $\delta_{\text{mse}}$ in (4). We empirically observed that kurtosis-based similarity was a good, fast approximation of MSE-based similarity, with a Spearman correlation of 0.87 on the BioGRID yeast dataset (Fig. 4a). Gemini is also based on the belief that similar networks reflect biological similarity. We considered the t-Stochastic Neighbor Embedding (t-SNE) (van der Maaten and Hinton 2008) of the human BioGRID network’s kurtosis representations, and compared these to the evidence type encoded in the GeneMANIA filenames (Mostafavi et al. 2008; Warde-Farley et al. 2010). We found that the t-SNE space largely clustered according to the type of network (Fig. 4b). We also found that the affinity-propagation-based clustering of kurtosis vectors had an adjusted Rand index (ARI) (Hubert and Arabie 1985) of 0.48 for the human dataset, compared to an ARI of 0 for random cluster assignments, indicating that similar kurtosis representations corresponded to similar biological evidence. Importantly, Gemini was able to automatically detect these similarities, both enabling networks with common patterns from different types of biological evidence to be grouped together and automatically separating different clusters of networks that may not be manually annotated with biologically relevant subtype labels.

We also evaluated the impact of the size *K* of the synthetic, mixed-up dataset of diffusion state matrices that Gemini generates as an intermediate step on the downstream protein function prediction performance. We expected that larger dataset sizes would improve downstream performance, but that smaller values of *K* would enable users with smaller computational budgets to compute vertex embeddings. If necessary, we can set K<M, reducing the number of networks in the SVD computation compared to the input dataset, and making Gemini run potentially faster than Mashup depending on the computational resources available. We varied *K* from 0.03125 to 2*M* and found that, as expected, larger values of *K* lead to better downstream performance for all species (Fig. 4c), although smaller values of *K* can still perform relatively well in resource-limited settings. For instance, the Gemini model with K=0.03125M (28 networks) on the human dataset has an $F_{1}$ score 89% of the model trained with K=M, and this increases to 95% with K=0.0625M (56 networks).

Finally, we analyzed the choice of kurtosis pooling by approximating network similarity using different moments. In addition to kurtosis, we compared protein prediction performance based on network similarity approximations using the first moment, with the mean of the diffusion distribution $\vec{r_{ik}}[j]$; the second central moment, with the variance of $\vec{r_{ik}}[j]$; and the third standardized moment, with the skew of $\vec{r_{ik}}[j]$. We found that kurtosis and skewness, the fourth and third standardized moments, consistently performed best on all species (Fig. 4d), and higher-order pooling significantly outperforms lower-order pooling (one-sided paired t-test *P* < 5e-5, comparing mean and variance pooling to skew and kurtosis pooling in terms of maximum F_1_ score). This validates our intuition that higher-order pooling can better capture both global and local network structure, compared to low-order pooling measures like mean and variance.

## 4 Conclusion

In this article, we have presented Gemini, a memory-efficient network integration method for large-scale and heterogeneous biological networks. We construct a kurtosis-based pooling of the diffusion state to cover different types and combinations of biological evidence, and use this vector to effectively weight each network. Although we address the under- and over-representation of different types of evidence, Gemini considers cluster membership, and therefore similarity, at the network-level. Future work could extend this notion to a vertex-based similarity, where two networks could be considered similar for some genes and different for others. Furthermore, as Gemini will weight unique networks more heavily than repetitive networks, outlier detection methods based on Gemini’s kurtosis-pooled network representations could be employed to remove particularly noisy or adversarial networks from consideration. Finally, future rigorous comparisons to supervised baselines across a variety of downstream tasks would further demarcate the benefit that Gemini and other unsupervised models can convey for gene network integration.

## Supplementary Material

btad247_Supplementary_Data

## Data Availability

The gene networks analyzed in this study and GOA labels used for evaluation are publically available from https://groups.csail.mit.edu/cb/mashup/mashup.tar.gz and https://stringdbstatic.org/download/protein.links.detailed.v11.5/10090.protein.links.detailed.v11.5.txt.gz. Data download and preprocessing scripts are available in our repository https://github.com/MinxZ/Gemini.
