# Supplementary material for: Gemini: memory-efficient integration of hundreds of gene networks with high-order pooling
Source: Bioinformatics. 2023 Jun 30;39(Suppl 1):i504–12. doi: 10.1093/bioinformatics/btad247 (PMC10311345; doi:10.1093/bioinformatics/btad247)
Supplement: btad247_Supplementary_Data [file btad247_supplementary_data.pdf]

# Supplemental Information

Gemini: Memory efficient integration of hundreds of gene networks with high-order pooling

A. Woicik, M. Zhang, H. Xu, S. Mostafavi, S. Wang

March 23, 2023

## S1 Network Statistics

We evaluate Gemini and other unsupervised, scalable network integration methods on two main network collections: the BioGRID [6] and STRING [8] databases. We downloaded the processed BioGRID networks processed from GeneMANIA [5, 9] for the mouse, human, and yeast gene networks. We download the processed STRING networks from Mashup [2], again focusing on the mouse, human, and yeast gene networks. Network statistics are shown in **Table S1**. We also show the distribution of gene vertex degrees for each dataset in **Figure S1**.

| Network Collection | Organism | Genes $n$ | Networks $M$ | Average network size | Average node degree |
|---|---|---|---|---|---|
| BioGRID | mouse | $n = 21{,}081$ | $M = 403$ | 10,283 | 23.7 |
| | human | $n = 19{,}695$ | $M = 895$ | 6802 | 18.0 |
| | yeast | $n = 6365$ | $M = 505$ | 1985 | 12.1 |
| STRING | mouse | $n = 21{,}104$ | $M = 6$ | 11,223 | 201.1 |
| | human | $n = 18{,}362$ | $M = 6$ | 7873 | 37.1 |
| | yeast | $n = 6311$ | $M = 6$ | 3221 | 42.1 |

Table S1: Network statistics for the BioGRID and STRING network collections. The average network size reports the average number of nodes contained in each network, while the number of genes $n$ is the number of unique genes contained across all $M$ networks of the collection. The average node degree is the number of nodes divided by the number of edge, averaged over all networks in the collection.

## S2 Evaluation and Statistical Tests

### S2.1 Comparison approaches

We benchmark Gemini against computational approaches that are able to scale to hundreds of gene networks. Given a collection of $M$ gene networks represented by their adjacency matrices $\{\mathbf{A}_i\}_{i=1}^{M}$, we include the Mashup [2] and BIONIC [3] network integration methods. Furthermore, we compute the average adjacency matrix of each collection as $\bar{\mathbf{A}} = \frac{1}{M}\sum_{i=1}^{M}\mathbf{A}_i$ and use this average representation to compute network embeddings with PCA, SVD, and Mashup (denoted Average Mashup).

**Mashup:** To facilitate a quality comparison, we set Gemini and Mashup to use the same model hyperparameters. Namely, we use the memory-efficient eigendecomposition Mashup framework, diffusion restart probability $\alpha = 0.5$, and gene embedding dimension of 200 for yeast and 400 for human and mouse. We re-implemented Mashup in python, and also used this framework to build Gemini.

**BIONIC:** We also compare to the BIONIC network integration approach for yeast networks, and for mouse and human STRING networks. For the yeast and human STRING network integration tasks, we used the default BIONIC repository configuration, including 3000 training epochs, a batch size of 2048, learning rate of 5e-4, a 64-dimension, 10 head, and 2-layer graph attention network (GAT), and embedding dimension of 512. For the mouse STRING integration task, we reduced the number of GAT layers to 1 in order to fit in GPU memory during inference, but otherwise retained the default hyperparameters. For all STRING integration tasks, we included all networks in each batch.

To train BIONIC on the BioGRID and STRING+BioGRID network integration collections, we reduced the size of the default architecture to fit in GPU memory and decreased the number of training epochs for a reasonable training speed. For the yeast BioGRID and STRING+BioGRID integrations we trained for 1000 epochs with

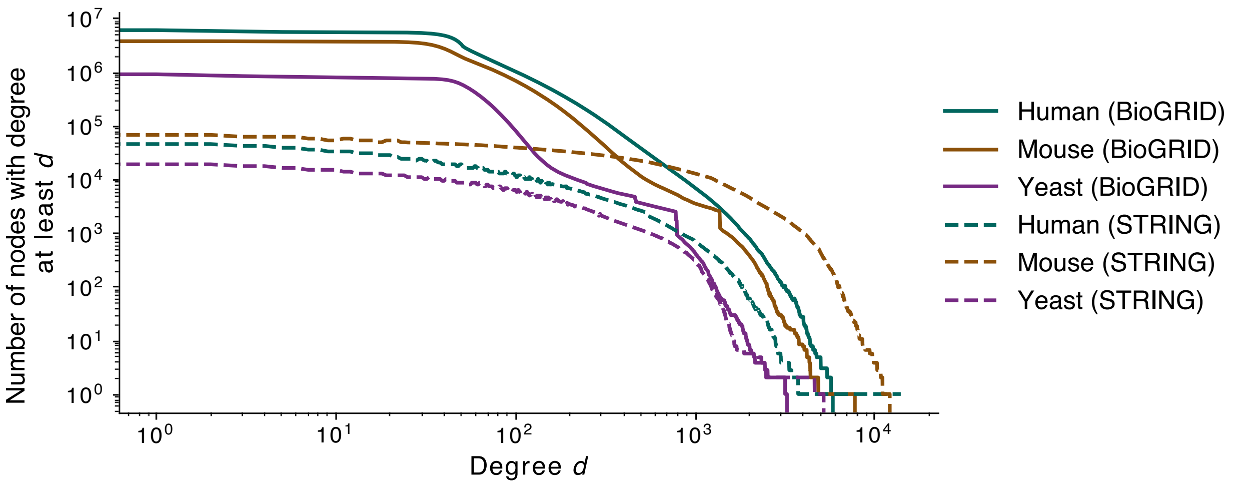


Figure S1: Number of nodes with degree at least $d$ for each of the network collections. The x-axis shows the range of node degrees in the dataset; the y-axis shows the number of nodes with degree at least $d = x$ in the given network collection, where each network's nodes are counted separately within a given dataset. BioGRID network statistics are plotted with solid lines while STRING network statistics are plotted with dashed lines; colors are used to differentiate human, mouse, and yeast datasets.

a batch size of 256 and learning rate of 5e-4. We reduced the GAT architecture to 64 dimension, 10 heads, and 1 layer, with an output embedding size of 200. We included 10 networks per batch. We attempted to train BIONIC on the mouse and human BioGRID collections, but were unable to train even a very minimal BIONIC architecture on an A4000 GPU (e.g., out-of-memory error for batch size of 128; GAT with 4 dimensions, 1 head, and 1 layer; output embedding dimension 8; 2 networks sampled per batch). These findings are also consistent with the scalability analysis conducted by BIONIC (Extended Data Figure 9 from **(author?)** [3]). We used the BIONIC pip package to train the BIONIC embeddings, providing the model and training details in a configuration file.

**PCA:** We take the average adjacency matrix $\bar{\mathbf{A}}$ of the input network collection and compute the top $k$ principal components as the output gene embeddings, with $k = 200$ for yeast and $k = 400$ for human and mouse.

**SVD:** We compute the truncated singular value decomposition of the average adjacency matrix $\mathbf{U\Sigma V}^\top = \bar{\mathbf{A}}$, with the output gene embedding matrix $\mathbf{V} \in \mathbb{R}^{n\times k}$, for $n$ the number of genes. Then, we take $k = 200$ for yeast and $k = 400$ for human and mouse.

**Average Mashup:** We also apply the Mashup method with eigendecomposition to the average adjacency matrix $\bar{\mathbf{A}}$. As in the full Mashup method, we use a diffusion restart probability $\alpha = 0.5$ and use a gene embedding dimension of 200 for yeast and 400 for human and mouse.

## S2.2 Evaluation

To evaluate the models, we compare the integrated gene embeddings' performance on a downstream protein function prediction task. We parameterize the downstream one-to-many classifier $f_\theta(\cdot)$ as a multi-layer perceptron with two hidden layers of 200- and 100-units respectively. Using the Gene Ontology Annotation (GOA) [1, 4] as ground-truth protein function labels, we perform 5-fold cross validation with 20% of the data used as the test split.

For each of the five test splits, we can compute the maximum $F_1$, macro-AUPRC, and micro-AUPRC. We can then compute the average and standard deviation over these five splits for each metric. When comparing Gemini's integrated embeddings for an organism and network collection to an existing approach, we then use a one-sided paired t-test to test whether Gemini outperforms the existing approach with respect to a given metric on the five test splits.

In some settings we further stratify the results by GO sub-ontology (**Fig. 2**, **Fig. S2**). To evaluate sub-ontology performance, we compute the maximum $F_1$, macro-AUPRC, and micro-AUPRC over all test splits when the output predictions are restricted to protein functions within the relevant sub-ontology. To compare different

methods' performance on a given sub-ontology, we again use a one-sided paired t-test for each metric on the five test splits when restricted to the sub-ontology of interest. **Table S2** shows the number of GO terms associated with each organism from each sub-ontology.

| Organism | BP terms | MF terms | CC terms |
|---|---|---|---|
| mouse | 12,306 | 3070 | 1250 |
| human | 15,418 | 4408 | 1829 |
| yeast | 4997 | 2416 | 974 |

Table S2: Number of GO terms for each organism by sub-ontology: Biological Process (BP), Molecular Function (MF), and Cellular Component (CC).

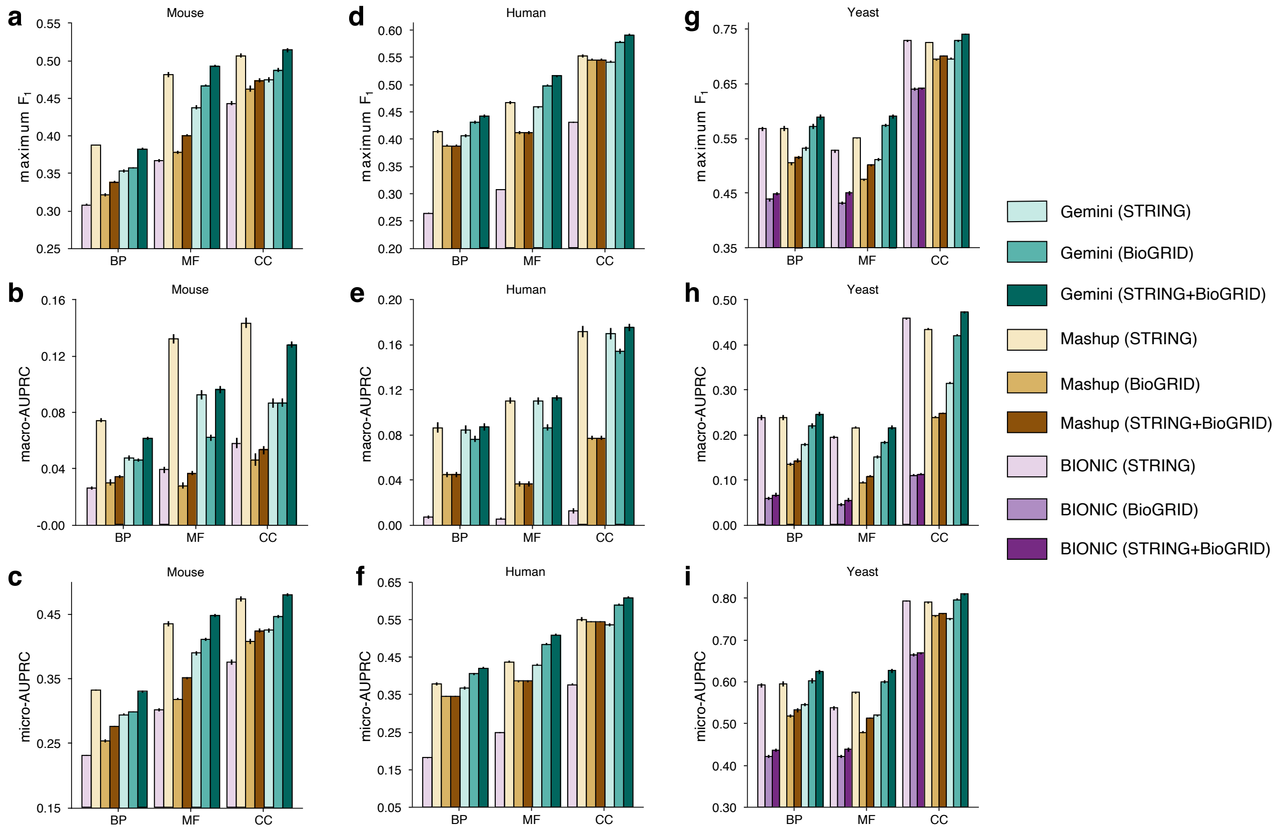


Figure S2: Comparison of network integration performance over different input network collections, stratified by GO sub-ontology. Performance of Gemini, Mashup, and BIONIC when applied to the STRING, BioGRID, and combined STRING+BioGRID network collection datasets for Mouse (a-c), Human (d-f), and Yeast (g-i) on a downstream protein function prediction task. Performance is measured by maximum $F_1$ score (a,d,g), macro-AUPRC (b,e,h), and micro-AUPRC(c,f,i). BIONIC has no results for mouse and human on BioGRID and STRING+BioGRID inputs due to GPU memory constraints. Higher values indicate better performance on all metrics; error bars denote standard error over the five test splits.

# S3 Computational Complexity

## S3.1 Gemini space and time complexity

First, we conduct a theoretical study of Gemini's time and space complexity. We divide Gemini into five main steps: diffusion, pooling, clustering, mixup, and decomposition.

In the diffusion step, we perform random walk with restart (RWR) for each network. The RWR steady state can be solved for using matrix inversion, which dominates the computational complexity of this step. For $M$ networks, each with $n$ genes, this step therefore takes $O(Mn^3)$ time and $O(Mn^2)$ space. In the pooling step, we compute the kurtosis over each node in each network. Kurtosis-pooling for each node requires computing the mean and standard deviation over a vector of length $n$. Therefore, we can complete the kurtosis-pooling

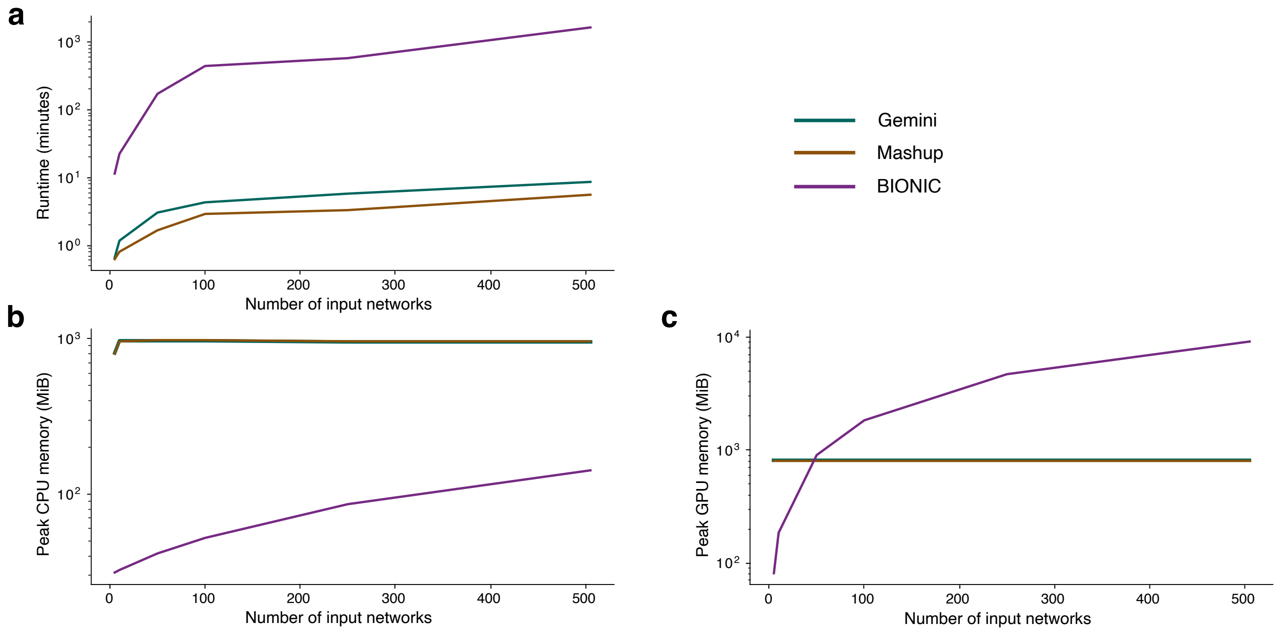


Figure S3: Network integration method performance across varied numbers of input networks from the yeast BioGRID network collection. We compare runtime (a), peak CPU memory utilization (MiB) and peak GPU memory utilization (MiB) of Gemini, Mashup, and BIONIC. For CPU and GPU utilization, Gemini and Mashup are practically indistinguishable.

step in $O(Mn^2)$ time and $O(Mn^2)$ space. In the clustering step, we use Affinity Propagation over the set of all networks as implemented in `sklearn` [7]. This has a time complexity of $O(M^2T)$, where $T = 200$ is the number of clustering iterations, and a space complexity $O(M^2)$. In the mixup step, we sample pairs of networks and then take computed weighted combination of the two diffusion state matrices. We repeat this mixup procedure $K$ times, requiring $O(Kn^2)$ time and $O(Kn^2)$ space. Finally, in the eigendecomposition step, we take the sum over the $K$ mixed-up networks transpose themselves, and then compute the eigenvectors, requiring $O(Kn^3)$ time and $O(n^2)$ space.

Overall, we can combine these steps to compute Gemini's overall time complexity as

$$O(Mn^3 + M^2T + Kn^3) = O(Mn^3 + Mn^2 + M^2T + Kn^2 + Kn^3) \tag{1}$$

and Gemini's overall space complexity as

$$O(Mn^2 + M^2 + Kn^2) = O(Mn^2 + Mn^2 + M^2 + Kn^2 + n^2). \tag{2}$$

In practice, we have $n \gg M$ for our networks and choose $K \leq M$ for our quantitiative experiments. Using the default $T = 200$ also results in $T < M \ll n$, so Gemini's computational complexity reduces to $O(Mn^3)$ time and $O(Mn^2)$ in these settings.

## S3.2 Ablation study comparison

To compare Gemini's time and memory usage in practice to other methods, we also conducted an ablation study in the number of networks. Here, we used the BioGRID yeast network collection and varied the number of input networks as $M \in [5, 10, 50, 100, 250, 505]$, where 505 is the full BioGRID dataset. We then compared the runtime of Gemini, Mashup, and BIONIC in these settings, keeping the model hyperparameters fixed to the BioGRID experiment hyperparameters. We also compared the cpu and gpu peak utilization of each method, as measured by `tracemalloc` and `torch.cuda.max_memory_allocated`. Ablation results are shown in **Fig. S3**. Due to time constraints, we trained BIONIC for a reduced number of epochs for the ablation and extrapolated the training time to the 1000 epochs that we would have trained BIONIC for; model inference time was measured separately and accounted for in the results.

# References


[1] Evelyn Camon, Michele Magrane, Daniel Barrell, Vivian Lee, Emily Dimmer, John Maslen, David Binns, Nicola Harte, Rodrigo Lopez, and Rolf Apweiler. The gene ontology annotation (GOA) database: sharing knowledge in uniprot with gene ontology. *Nucleic Acids Res.*, 32(Database issue):D262–6, January 2004.

[2] Hyunghoon Cho, Bonnie Berger, and Jian Peng. Compact integration of Multi-Network topology for functional analysis of genes. *Cell Syst*, 3(6):540–548.e5, December 2016.

[3] Duncan T Forster, Sheena C Li, Yoko Yashiroda, Mami Yoshimura, Zhijian Li, Luis Alberto Vega Isuhuaylas, Kaori Itto-Nakama, Daisuke Yamanaka, Yoshikazu Ohya, Hiroyuki Osada, Bo Wang, Gary D Bader, and Charles Boone. BIONIC: biological network integration using convolutions. *Nat. Methods*, 19(10):1250–1261, October 2022.

[4] Rachael P Huntley, Tony Sawford, Prudence Mutowo-Meullenet, Aleksandra Shypitsyna, Carlos Bonilla, Maria J Martin, and Claire O'Donovan. The GOA database: Gene ontology annotation updates for 2015. *Nucleic Acids Res.*, 43(D1):D1057–D1063, November 2014.

[5] Sara Mostafavi, Debajyoti Ray, David Warde-Farley, Chris Grouios, and Quaid Morris. GeneMANIA: a real-time multiple association network integration algorithm for predicting gene function. *Genome Biol.*, 9 Suppl 1:S4, June 2008.

[6] Rose Oughtred, Jennifer Rust, Christie Chang, Bobby-Joe Breitkreutz, Chris Stark, Andrew Willems, Lorrie Boucher, Genie Leung, Nadine Kolas, Frederick Zhang, Sonam Dolma, Jasmin Coulombe-Huntington, Andrew Chatr-Aryamontri, Kara Dolinski, and Mike Tyers. The BioGRID database: A comprehensive biomedical resource of curated protein, genetic, and chemical interactions. *Protein Sci.*, 30(1):187–200, January 2021.

[7] F Pedregosa, G Varoquaux, A Gramfort, V Michel, B Thirion, O Grisel, M Blondel, P Prettenhofer, R Weiss, V Dubourg, J Vanderplas, A Passos, D Cournapeau, M Brucher, M Perrot, and E Duchesnay. Scikit-learn: Machine learning in Python. *J. Mach. Learn. Res.*, 2011.

[8] Damian Szklarczyk, Annika L Gable, David Lyon, Alexander Junge, Stefan Wyder, Jaime Huerta-Cepas, Milan Simonovic, Nadezhda T Doncheva, John H Morris, Peer Bork, Lars J Jensen, and Christian von Mering. STRING v11: protein-protein association networks with increased coverage, supporting functional discovery in genome-wide experimental datasets. *Nucleic Acids Res.*, 47(D1):D607–D613, January 2019.

[9] David Warde-Farley, Sylva L Donaldson, Ovi Comes, Khalid Zuberi, Rashad Badrawi, Pauline Chao, Max Franz, Chris Grouios, Farzana Kazi, Christian Tannus Lopes, Anson Maitland, Sara Mostafavi, Jason Montojo, Quentin Shao, George Wright, Gary D Bader, and Quaid Morris. The GeneMANIA prediction server: biological network integration for gene prioritization and predicting gene function. *Nucleic Acids Res.*, 38(Web Server issue):W214–20, July 2010.
